# Supplementary material for: Vaccine co-display of CSP and Pfs230 on liposomes targeting two Plasmodium falciparum differentiation stages
Source: Commun Biol. 2022 Aug 1;5:773. doi: 10.1038/s42003-022-03688-z (PMC9341416; doi:10.1038/s42003-022-03688-z)
Supplement: Supplementary file 2 — Supplemental Information [file 42003_2022_3688_MOESM2_ESM.pdf]

Supplementary data

Vaccine Co-display of CSP and Pfs230 on Liposomes Targeting Two *Plasmodium falciparum* Differentiation Stages

Wei-Chiao Huang, Moustafa Mabrouk, Luwen Zhou, Minami Baba, Mayumi Tachibana, Motomi Torii, Eizo Takashima, Emily Locke, Jordan Plieskatt, C. Richter King, Camila H. Coelho, Patrick E. Duffy, Carole Long, Takafumi Tsuboi, Kazutoyo Miura, Yimin Wu, Tomoko Ishino*, Jonathan Lovell*

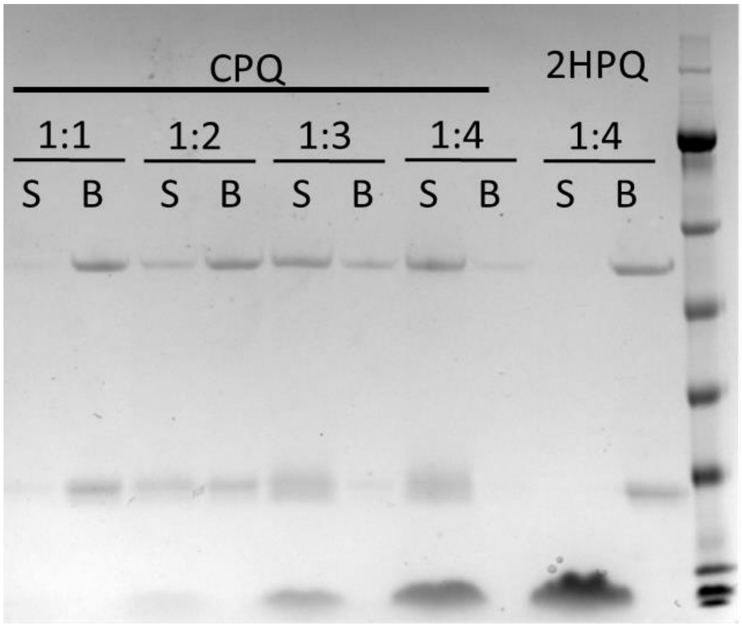

Supplementary Figure S1. Full gel blot showing antigen binding to CP liposomes was measured by Ni-NTA bead competition assay (shown in Fig 1b). Identical PoP liposomes lacking cobalt served as a control. The bands at the bottom of the gel corresponds to the CoPoP or PoP lipid itself.

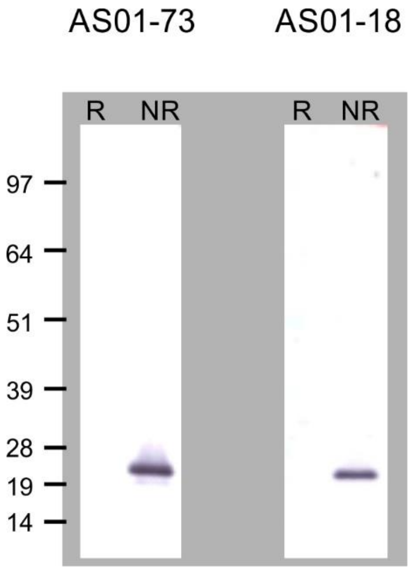

Supplementary Figure S2. Western blot with two human anti-Pfs230 mAbs AS01-73 and AS01-18 mAb (10 mg/lane) were tested with Pfs230D1 protein (20 ng/lane) both under reducing (R) and non-reducing (NR) conditions. The molecular weight of Pfs230D1 recombinant protein is ~21 kDa.

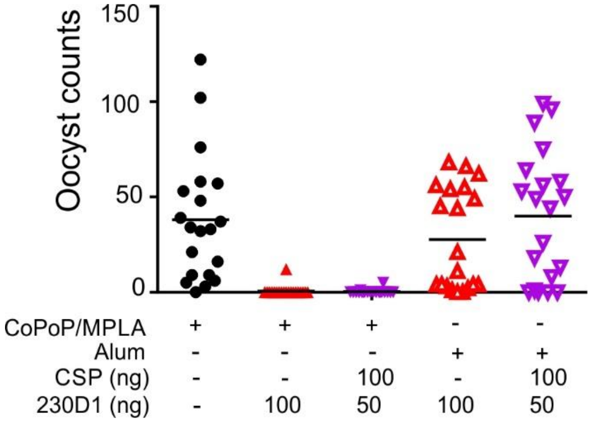

Supplementary Figure S3. SMFA functional assay. (**a**) Outbred Mice immunized with CSP, Pfs230D1+ or bivalent antigens with CoPoP liposomes or Alum adjuvant on day 0 and day 21.

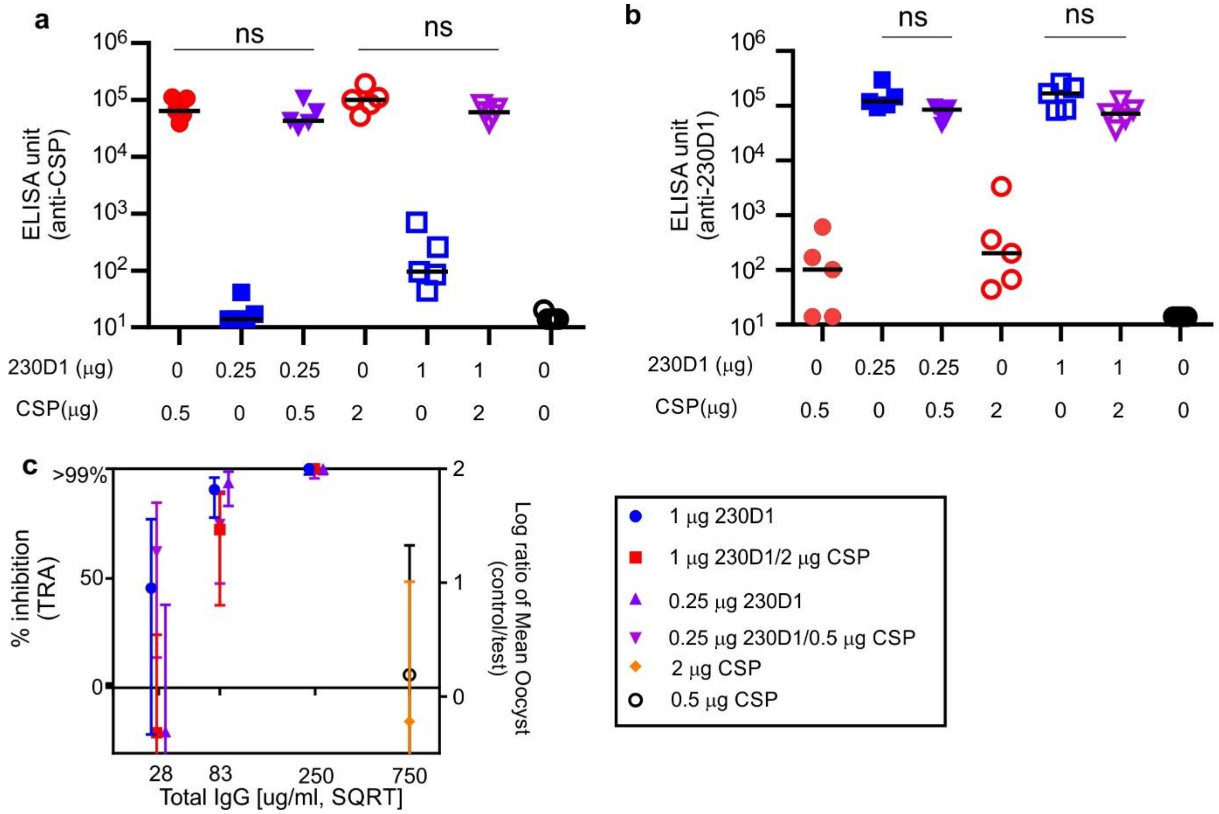

Supplementary Figure S4. Duplex immunization with CSP and 230 in ICR mice. Mice were immunized with CSP, Pfs230D1+ or duplex antigens with CoPoP/PHAD/QS-21 liposomes on day 0 and day 21, and final bleedings were collected on day 42. **(a)** Anti-CSP ELISA units and **(b)** Anti-230D1+ ELISA units. Purified IgGs were fed to n=20 mosquitoes per group at the indicated IgG concentration in the standard membrane feeding assay (SMFA) and oocysts were counted 8 days later. Transmission-reducing activity corresponds to the reduction of oocyst number compared to the adjuvant alone control. The error bar indicates 95 % confidence intervals. Experiments were performed with n=5 of individual mice and the lines in **A** and **B** represent geometric mean. One-way ANOVA followed by Tukey test using log transformed data were used to compare differences among groups immunized with homologous antigens. Statistical analysis is further reported in Supplementary Table S5.

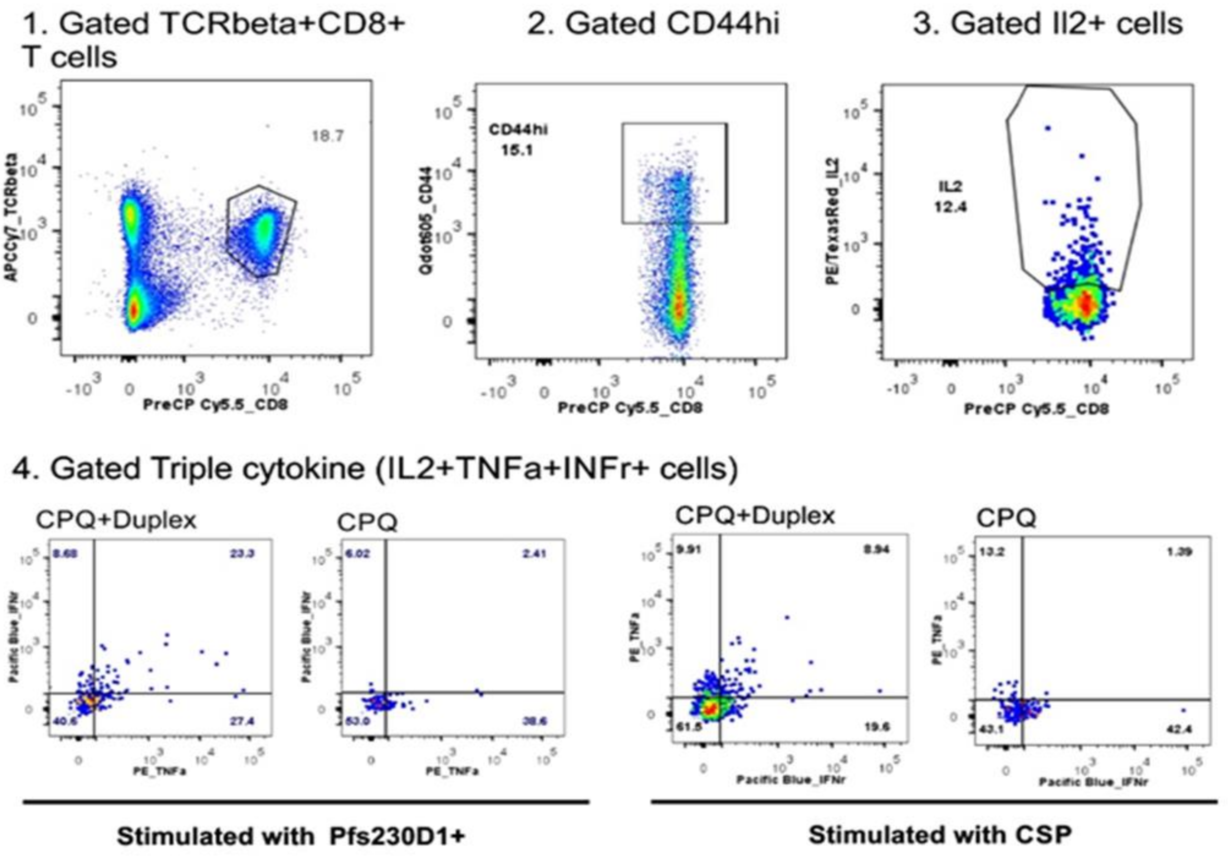

Supplementary Figure S5. Flow gating for splenocytes collected from mice immunized with CSP (100 ng)/ 230 (50 ng). Splenocytes were stimulated with individual antigens (1 µg/ml) for 18 hr, and $IL2^+TNFa^+IFN\text{-}\gamma^+$ triple positive $CD8^+$ T cells were gated.

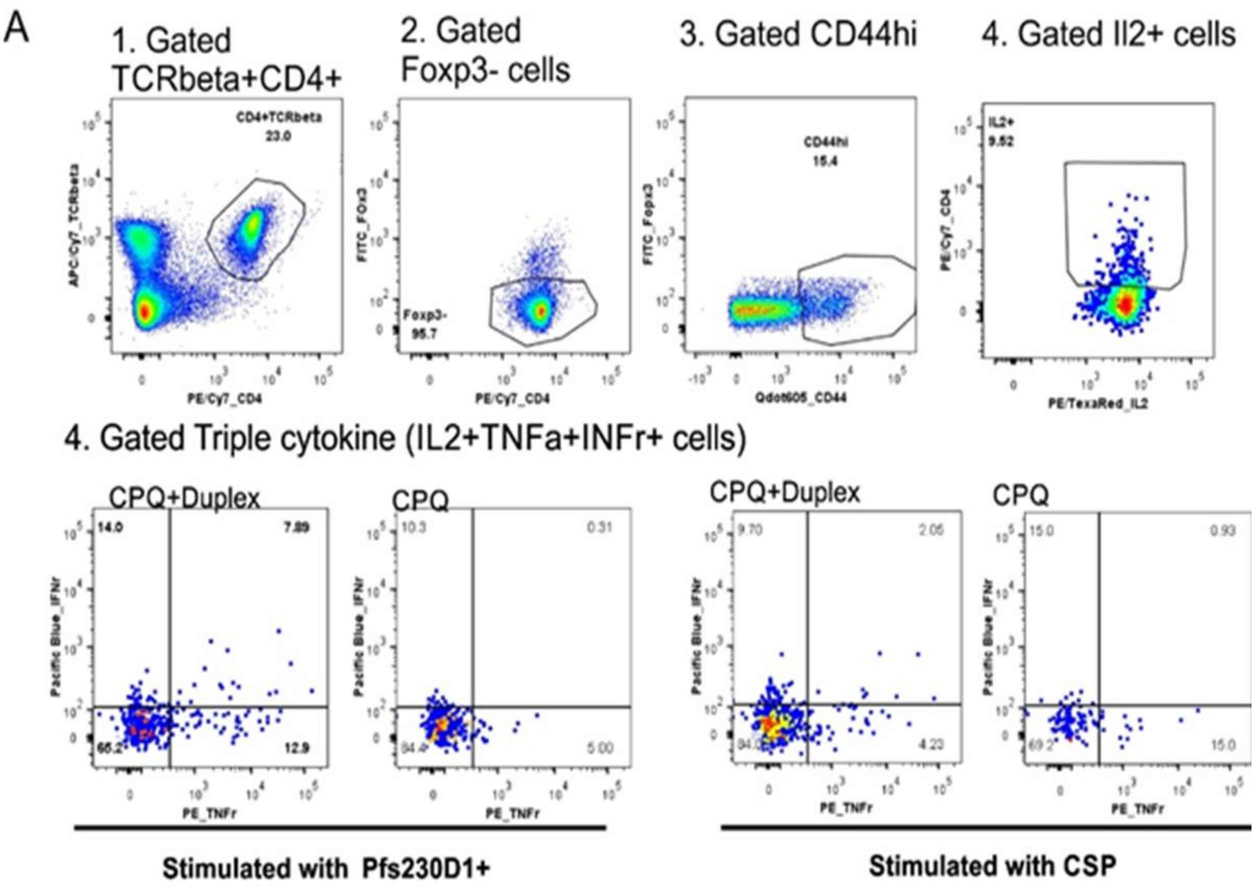

Supplementary Figure S6. Flow gating for splenocytes collected from mice immunized with CSP (100 ng)/ 230 (50 ng) with indicated adjuvants. Splenocytes were stimulated with individual antigens (1 ug/ml) for 18 hr, and $IL2^{+}TNFa^{+}IFN\text{-}\gamma^{+}$ triple positive $CD4^{+}$ T cells were gated.

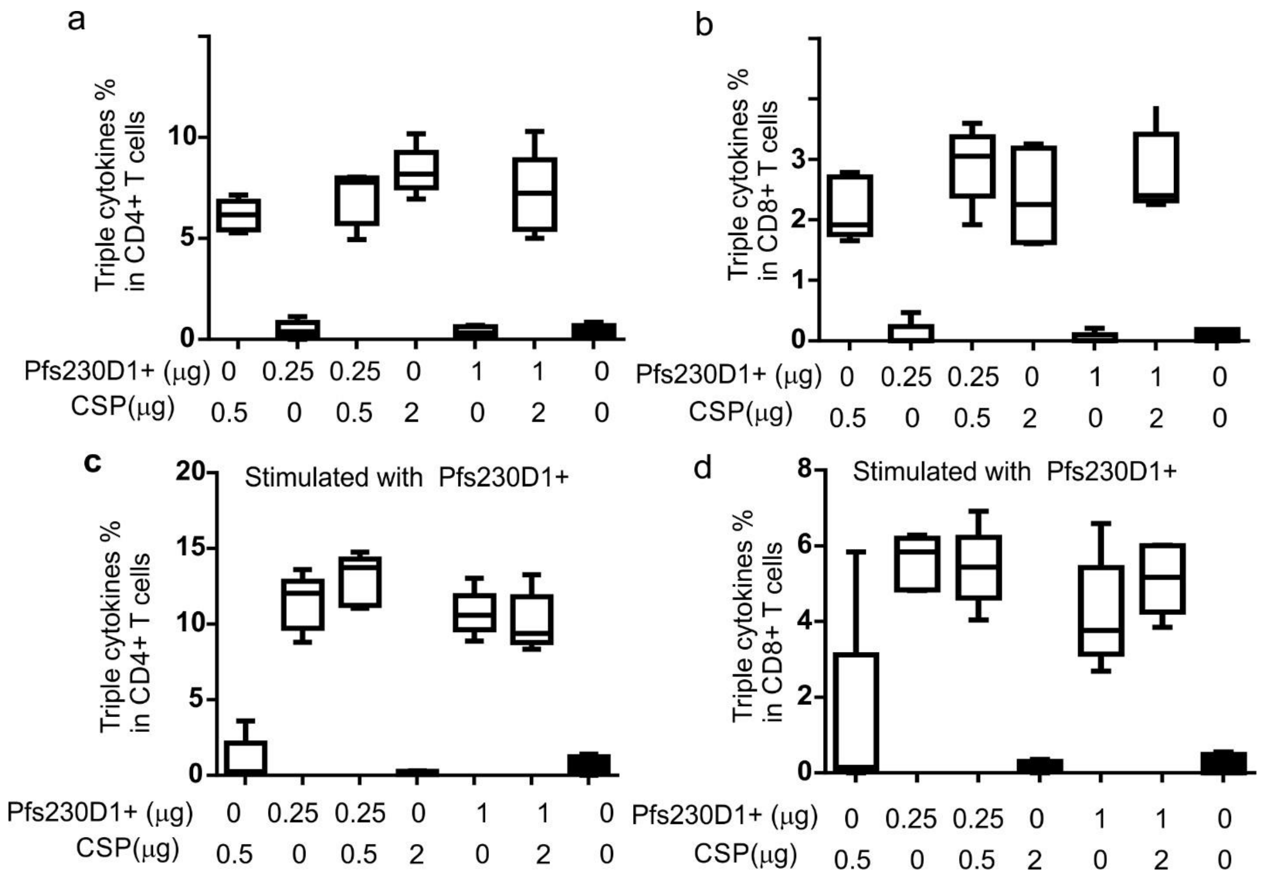

Supplementary Figure S7. Polyfunctional antigen-specific T cells in splenocytes collected from outbred mice immunized with CSP /230 with CPQ using indicated dose on day 0 and day 21 with spleen collection on day 42. Splenocytes were stimulated with individual antigens (1 μg/ml) for 18 hr, and $IL2^{+}TNFa^{+}IFN\text{-}\gamma^{+}$ triple positive T cell population were gated. (**a**) $CD4^{+}$ T cells or (**b**) $CD8^{+}$ T cells positive for triple cytokine gating, stimulated with CSP. (**c**) $CD4^{+}$ T cells or (**d**) $CD8^{+}$ T cells positive for triple cytokine gating strategy, stimulated with Pfs230D1+. The line shows the median value, box shows the interquartile range and whiskers represent the data range.

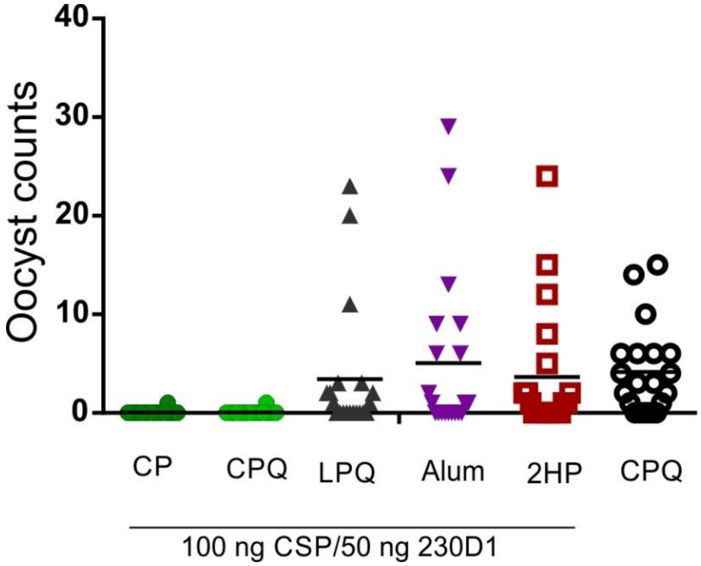

Supplementary Figure S8. SMFA functional assay. C57Bl/6 mice immunized with CSP (50 ng)/ Pfs230D1+ (50 ng) on day 0 and day 21 with indicated adjuvants.

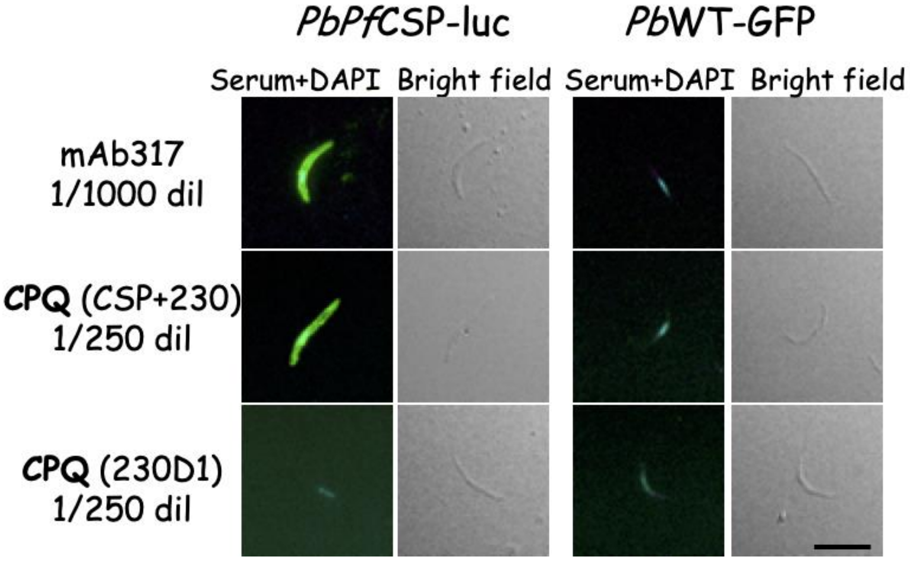

Supplementary Figure S9. IFA images using diluted post immune sera for PbPfCSP-luc sporozoite and PbWT sporozoite. Scale bar: 5 µm.

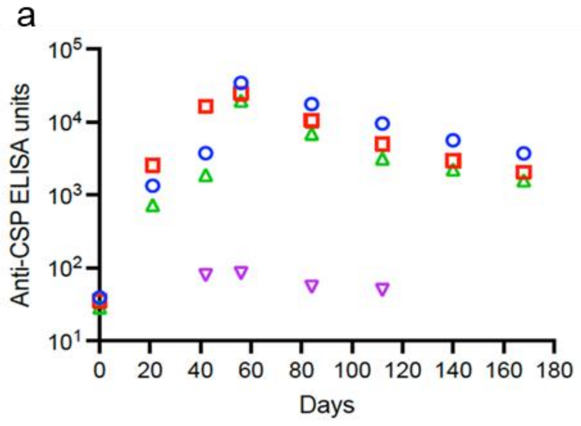

Supplementary Figure S10. Bivalent CSP and Pfs230D1+ immunization of rabbits. Rabbits received 3 intramuscular immunizations on day 0, 21 and 42. On day 0, 21, 42, 56, 84, 112, 140, and 168 (**a**) anti-CSP and (**b**) anti-Pfs230D1+ ELISA units were measured. For **a** and **b** data points represents geometric mean of 3 female and 3 male rabbits in each group. Blue circles indicate bivalent, liposome-displayed CSP/Pfs230D1+. Red squares indicate bivalent CSP/Pfs230D1+ formulated with Alum. Green triangles indicate monovalent, liposome-displayed CSP. Purple triangles indicate monovalent, liposome-displayed Pfs230D1+. For monovalent vaccine groups, ELISA units against homologous protein were evaluated for all time points, while only for days 42, 56, 84 and 112 against heterologous protein.

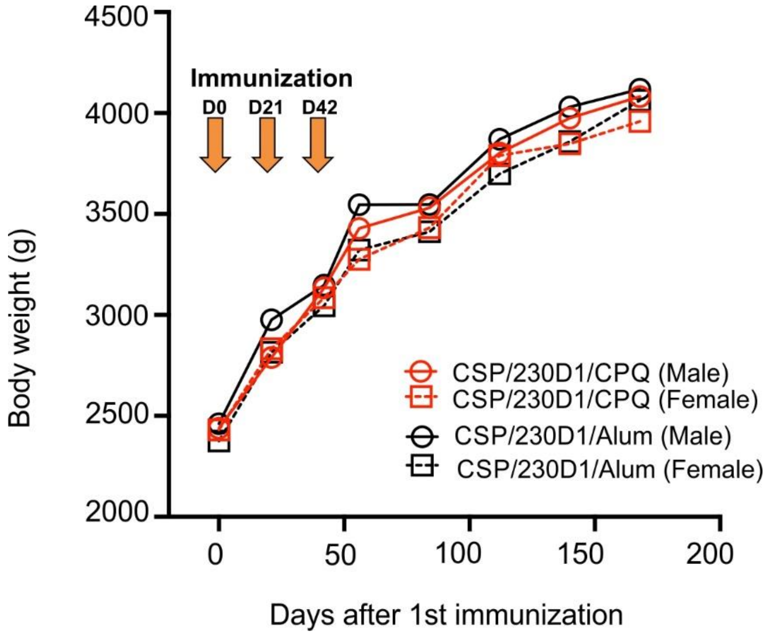

Supplementary Figure S11. Rabbit immunization with CSP (20 µg)/ Pfs230D1+ (10 µg) with CPQ on day 0, 21 and 42. The weight of each rabbit was tracked over time. Data show no loss of weight after immunization.

Supplementary Table S1: Sequences of antigens used in this study (not including C-terminus his tag)

CSP sequence (a.a. 27-383)	GSSSNTRVLNELNYDNAGTNLYNELEMNYYGKQENWYSLKKNSRSLGENDDGNNEDNEK LRKPKHKKLKQPADGNPDPNANPNVDPNANPNVDPNANPNVDPNANPNANPNANPNANP NANPNANPNANPNANPNANPNANPNANPNANPNANPNANPNANPNANPNANPNVDPNAN PNANPNANPNANPNANPNANPNANPNANPNANPNANPNANPNANPNANPNANPNANPNA NPNANPNANPNKNNQGNGQGHNMPNDPNRNVDENANANSAVKNNNNEEPSDKHIKEYLN KIQNSLSTEWSPCSVTCGNGIQVRIKPGSANKPKDELDYANDIEKKICKMEKCSSVFNV VNS
Pfs230D1+ sequence (a.a. 552-731)	VGVDELDKIDLSYETTESGDTAVSEDSYDKYASNNTNKEYVCDFTDQLKPTESGPKVKK CEVKVNEPLIKVKIICPLKGSVEKLYDNIEYVPKKSPYVVLTKEETKLKEKLLSKLIYG LLISPTVNEKENNFKEGVIEFTLPPVVHKATVFYFICDNSKTEDDNKKGNRGIVEVYVE PYG

Supplementary Table S2. Statistical analysis tests of log transformed titer data from outbred mice immunized on day 0, day 21 and final bleeding on day 42. Antigens are at nanogram dose. Mean values are not included here for simplicity. $^{**}p<0.005$ $^{***}p<0.0005$, $^{****}p<0.0001$

One-way ANOVA followed by Tukey test	Anti-CSP titer Adjusted P Value	Anti-230 titer Adjusted P Value
200ng CSP/CP vs. 100 ng CSP, 50 ng 230/CP	*ns*	--
200ng CSP/CP vs. 200 ng CSP/Alum	***	--
200ng CSP/CP vs. 100 ng CSP,50 ng 230/Alum	****	--
100 ng 230/CP vs.100 ng CSP, 50 ng 230/CP	****	*ns*
100ng 230/CP vs. 100 ng 230/Alum	--	****
100ng 230/CP vs. 100 ng CSP,50 ng 230/Alum	--	****
100 ng CSP, 50 ng 230/CP vs. 200 ng CSP/Alum	***	****
100 ng CSP, 50 ng 230/CP vs. 100 ng 230/Alum	--	**
100 ng CSP, 50 ng 230/CP vs. 100 ng CSP, 50 ng 230/Alum	****	--
200 ng CSP/Alum vs. 100 ng CSP, 50 ng 230/Alum	*ns*	--
100 ng 230/Alum vs. 100 ng CSP, 50 ng 230/Alum	--	**

Supplementary Table S3. SMFA data. Each SMFA test group (i.e., each table entry) was assessed with n=20 mosquitos per group using purified IgGs from pooled serum of n=5-10 mice. Table entries indicate the sample dose used for immunization. Statistical testing is based on a zero-inflated negative binomial random effects model.

	**Sample name**	IgG conc [ug/ml]	Mean oocyst	mosquitoes[d]	% inhibition (TRA)			
					estimate	95%CI Lo	95%CI Hi	p-value
SMFA# 276-1[a]	Normal mouse Ab	750	27.0	36/40				
	CP alone	750	38.0	19/20	-40.6	-189.0	34.6	0.366
	100 ng 230/CP	750	0.6	1/20	97.8	95.3	99.0	0.001
	100 ng CSP/ 50 ng 230/CP	750	0.3	2/20	98.9	97.1	99.7	0.001
	100 ng 230/Alum	750	27.7	18/20	-2.3	-115.8	54.6	0.990
	100 ng CSP/ 50 ng 230/Alum	750	40.0	16/20	-47.8	-213.9	31.8	0.309
SMFA# 313[b]	Normal mouse Ab	750	8.3	22/33				
	100 ng CSP/ 50 ng 230/CP	750	0.1	1/20	99.4	98.1	99.9	0.001
	100 ng CSP/ 50 ng 230/CPQ	750	0.1	1/20	99.4	97.3	99.8	0.001
	100 ng CSP/ 50 ng 230/LPQ	750	3.4	10/20	58.9	7.8	81.4	0.032
	100 ng CSP/ 50 ng 230/Alum	750	5.1	11/20	39.0	-41.1	73.8	0.219
	100 ng CSP/ 50 ng 230/2HP	750	3.7	11/20	55.9	7.2	80.8	0.035
	CPQ alone	750	4.2	15/20	49.8	-17.5	79.9	0.112
SMFA# 318[c]	Normal mouse Ab	750	6.3	30/40				
	CPQ alone	750	11.2	15/20	-76.3	-277.6	19.0	0.154
	2 µg CSP/CPQ	750	7.3	11/20	-15.4	-148.4	48.4	0.743
	1 µg 230/CPQ	250	0.0	0/20	100.0	97.5	100.0	0.001
		83	0.6	5/20	90.5	77.8	96.0	0.001
		28	3.5	11/20	45.5	-21.3	77.0	0.139
	2 µg CSP+1 µg 230/CPQ	250	0.0	0/20	100.0	98.4	100.0	0.001
		83	1.8	4/20	72.3	37.6	88.5	0.003
		28	10.6	13/20	-67.6	-251.7	24.3	0.190
	0.5 µg CSP/CPQ	750	6.0	13/20	5.9	-148.6	65.0	0.849
	0.25 µg 230/CPQ	250	0.0	0/20	100.0	97.6	100.0	0.001
		83	0.4	4/20	93.7	83.1	98.7	0.001
		28	8.8	16/20	-39.1	-214.5	37.9	0.400
	0.5 µg CSP+0.25 µg 230/CPQ	250	0.1	1/20	98.4	95.6	99.8	0.001
		83	1.6	10/20	74.7	47.6	89.6	0.001
		28	2.4	9/20	62.1	13.8	84.5	0.013

[a] SMFA 276-1, n=10 outbred mice pooled sera were used for each group.
[b] SMFA 313, n=6 B6 mice pooled sera were used for each group.
[c] SMFA 318, n=5 B6 mice pooled sera were used for each group.
[d] Number of infected mosquitoes / Number of dissected mosquitoes

Supplementary Table S4. Statistical analysis tests of log transformed titer data from C57BL/6 mice immunized on day 0 and 21 and bled on day 42. Dual antigens at 100 ng CSP, 50 ng 230D1 admixed with indicated adjuvants (CP, CPQ, LPQ, 2HP, Alum). N=6 B6 mice per group. $^{*}p<0.05$, $^{**}p<0.005$, $^{***}p<0.0005$, $^{****}p<0.0001$

One-way ANOVA followed by Tukey test	Anti-CSP titer Adjusted P Value	Anti-230D1 titer Adjusted P Value
Duplex/CP vs. Duplex/ CPQ_	*ns*	*ns*
Duplex/CP vs. Duplex/LPQ	***	****
Duplex/CP vs. Duplex/Alum	****	**
Duplex/CP vs. Duplex/2HP	****	****
Duplex/CP vs. Control	****	****
Duplex/CPQ vs. Duplex/LPQ	***	****
Duplex/CPQ vs. Duplex/Alum	****	****
Duplex/CPQ vs. Duplex/2HP	****	****
Duplex/CPQ vs. Control	****	****
Duplex/LPQ vs. Duplex/Alum	*	*ns*
Duplex/LPQ vs. Duplex/2HP	*ns*	*ns*
Duplex/LPQ vs. Control	****	****
Duplex/Alum vs. Duplex/2HP	*ns*	*ns*
Duplex/Alum vs. Control	****	****
Duplex/2HP vs. Control	****	****

Supplementary Table S5. Statistical analysis tests of log transformed titer data from outbred mice immunized on day0, day 21 and final bleeding on day 42. CSP (high dose) represents 2 μg CSP/CPQ, 230D1 (high dose) represents 1 μg 230D1/CPQ, CPS/230 (high dose) represents 2 μg CSP+1 μg 230D1/CPQ, CPS (low dose) represents 0.5 μg CSP/CPQ, 230 (low dose) represents 0.25 μg 230/CPQ, CPS/230 (low dose) represents 0.5 μg CSP+0.25 μg 230/CPQ and control represents CPQ alone. Mean values are not included in the table.

One-way ANOVA followed by Tukey test	Anti-CSP titer Adjusted P Value	Anti-230 titer Adjusted P Value
CSP (high dose) vs. CSP/230 (high dose)	0.2858	--
CSP (high dose) vs CSP (low dose)	0.5617	--
CSP (high dose) vs. CSP/230 (low dose)	0.1082	--
230 (high dose) vs. CSP/230 (high dose)	--	0.0815
230 (high dose) vs. 230 (low dose)	--	0.9977
230 (high dose) vs. CSP/230D1 (low dose)	--	0.0935
CSP/230 (high dose)vs. CSP (low dose)	0.9506	--
CSP/230 (high dose)vs. 230 (low dose)	--	0.1134
CSP/230 (high dose) vs. CSP/230 (low dose)	0.9333	0.9998
CSP (low dose) vs. CSP/230 (low dose)	0.6824	--
230 (low dose) vs. CSP/230 (low dose)	--	0.1294

Supplementary Table S6. Statistical analysis test using log transformed titer data from C57BL/6 mice immunized on day 0, day 14 and day 28, and final bleeding on day 42. CSP (high dose) represents 2 μg CSP/CPQ, 230 (high dose) represents 1 μg 230/CPQ, CPS/230 (high dose) represents 2 μg CSP+1 μg 230/CPQ, CPS (low dose) represents 0.5 μg CSP/CPQ, 230 (low dose) represents 0.25 μg 230/CPQ, CPS/230 (low dose) represents 0.5 μg CSP+0.25 μg 230/CPQ and control represents CPQ alone. $^{*}p<0.05$, $^{**}p<0.005$, $^{***}p<0.0005$, $^{****}p<0.0001$

One-way ANOVA followed by Tukey test	Anti-CSP titer	Anti-230D1 titer
Tukey's multiple comparisons test	Adjusted P Value	Adjusted P Value
CSP/230 (high dose) vs. CSP/230 (low dose)	*ns*	*ns*
CSP/230 (high dose) vs. CSP (high dose)	*	****
CSP/230 (high dose) vs. CSP (low dose)	****	****
CSP/230 (high dose) vs. 230 (high dose)	****	*ns*
CSP/230 (high dose) vs. CPQ alone	****	****
CSP/230 (high dose) vs. no treatment	****	****
CSP/230 (low dose) vs. CSP (high dose)	*ns*	****
CSP/230 (low dose) vs. CSP (low dose)	*	****
CSP/230 (low dose) vs. 230 (high dose)	****	*ns*
CSP/230 (low dose) vs. CPQ alone	****	****
CSP/230 (low dose) vs. no treatment	****	****
CSP (high dose) vs. CSP (low dose))	*ns*	*ns*
CSP (high dose) vs. 230 (high dose)	****	****
CSP (high dose) vs. CPQ alone	****	*ns*
CSP (high dose) vs. no treatment	****	*ns*
CSP (low dose) vs. 230 (high dose)	****	****
CSP (low dose) vs. CPQ alone	****	*ns*
CSP (low dose) vs. no treatment	****	*ns*
230 (high dose) vs. CPQ alone	*ns*	****
230 (high dose) vs. no treatment	*ns*	****
CPQ alone vs. no treatment	*ns*	*ns*
