# Supplementary material for: Vaccine co-display of CSP and Pfs230 on liposomes targeting two Plasmodium falciparum differentiation stages
Source: Commun Biol. 2022 Aug 1;5:773. doi: 10.1038/s42003-022-03688-z (PMC9341416; doi:10.1038/s42003-022-03688-z)
Supplement: Supplementary file 3 — NR Reporting Summary [file 42003_2022_3688_MOESM3_ESM.pdf]

nature portfolio

Corresponding author(s): Lovell
Last updated by author(s): 06092022

Reporting Summary

Nature Portfolio wishes to improve the reproducibility of the work that we publish. This form provides structure for consistency and transparency in reporting. For further information on Nature Portfolio policies, see our Editorial Policies and the Editorial Policy Checklist.

Statistics

For all statistical analyses, confirm that the following items are present in the figure legend, table legend, main text, or Methods section.

n/a	Confirmed	
☐	☒	The exact sample size (n) for each experimental group/condition, given as a discrete number and unit of measurement
☐	☒	A statement on whether measurements were taken from distinct samples or whether the same sample was measured repeatedly
☐	☒	The statistical test(s) used AND whether they are one- or two-sided<br>*Only common tests should be described solely by name; describe more complex techniques in the Methods section.*
☒	☐	A description of all covariates tested
☐	☒	A description of any assumptions or corrections, such as tests of normality and adjustment for multiple comparisons
☐	☒	A full description of the statistical parameters including central tendency (e.g. means) or other basic estimates (e.g. regression coefficient) AND variation (e.g. standard deviation) or associated estimates of uncertainty (e.g. confidence intervals)
☒	☐	For null hypothesis testing, the test statistic (e.g. F, t, r) with confidence intervals, effect sizes, degrees of freedom and P value noted<br>*Give P values as exact values whenever suitable.*
☒	☐	For Bayesian analysis, information on the choice of priors and Markov chain Monte Carlo settings
☒	☐	For hierarchical and complex designs, identification of the appropriate level for tests and full reporting of outcomes
☒	☐	Estimates of effect sizes (e.g. Cohen's d, Pearson's r), indicating how they were calculated

Our web collection on statistics for biologists contains articles on many of the points above.

Software and code

Policy information about availability of computer code

Data collection	No code for data collection was used.
Data analysis	Excel and GraphPad Prism were used for data analysis and plotting. FlowJo was used for flow analysis. Image Lab software was used for SDS PAGE and slot blot. Coreldraw was used to arrange figures.

For manuscripts utilizing custom algorithms or software that are central to the research but not yet described in published literature, software must be made available to editors and reviewers. We strongly encourage code deposition in a community repository (e.g. GitHub). See the Nature Portfolio guidelines for submitting code & software for further information.

Data

Policy information about availability of data

All manuscripts must include a data availability statement. This statement should provide the following information, where applicable:

- Accession codes, unique identifiers, or web links for publicly available datasets
- A description of any restrictions on data availability
- For clinical datasets or third party data, please ensure that the statement adheres to our policy

All raw data are available upon request.

March 2021

Human research participants

Policy information about studies involving human research participants and Sex and Gender in Research.

Reporting on sex and gender	*Use the terms sex (biological attribute) and gender (shaped by social and cultural circumstances) carefully in order to avoid confusing both terms. Indicate if findings apply to only one sex or gender; describe whether sex and gender were considered in study design whether sex and/or gender was determined based on self-reporting or assigned and methods used. Provide in the source data disaggregated sex and gender data where this information has been collected, and consent has been obtained for sharing of individual-level data; provide overall numbers in this Reporting Summary. Please state if this information has not been collected. Report sex- and gender-based analyses where performed, justify reasons for lack of sex- and gender-based analysis.*
Population characteristics	*Describe the covariate-relevant population characteristics of the human research participants (e.g. age, genotypic information, past and current diagnosis and treatment categories). If you filled out the behavioural & social sciences study design questions and have nothing to add here, write "See above."*
Recruitment	*Describe how participants were recruited. Outline any potential self-selection bias or other biases that may be present and how these are likely to impact results.*
Ethics oversight	*Identify the organization(s) that approved the study protocol.*

Note that full information on the approval of the study protocol must also be provided in the manuscript.

Field-specific reporting

Please select the one below that is the best fit for your research. If you are not sure, read the appropriate sections before making your selection.

☒ Life sciences ☐ Behavioural & social sciences ☐ Ecological, evolutionary & environmental sciences

For a reference copy of the document with all sections, see nature.com/documents/nr-reporting-summary-flat.pdf

Life sciences study design

All studies must disclose on these points even when the disclosure is negative.

Sample size	For in vivo studies, sample sizes are indicated in the methods and/or figures captions. For mouse immunization and functional assays, most groups used n=10 mice. For mouse immunization and functional assays, each group used n=10 mice for Fig 4 to compared individual antigen and bivalent antigens with CoPoP liposomes or Alum in outbred mice. For mouse immunization and functional assays with C57Bl/6 mice, n=6 mice were used to compared bivalent antigens with CoPoP liposomes or other adjuvants (Fig 5). Mice challenge study were measured using n=6 C57Bl/6 mice for bivalent antigens or CSP antigen with CoPoP liposomes and n=3 C57Bl/6 mice for control (CoPoP liposomes and Pfs230D1+ with CoPoP liposomes). For rabbit immunization studies, groups contained n=6 rabbits per group.
Data exclusions	No data were excluded.
Replication	Antigen/liposomes binding assays in this study correspond to separately setup experiments. Serum from immunized mice has been checked by two independent labs after shipping (NIAID and SUNY at Buffalo).ELISA results were consistent in two independent labs .
Randomization	There was no randomization in this experiments, beyond randomly grouping mice into varying groups without a formal protocol. Blinding was not used in our experiments.
Blinding	ELISA and SMFA results were performed in the NIAID lab using shipped serum, with minimal knowledge of the samples, however it was not blinded.

Reporting for specific materials, systems and methods

We require information from authors about some types of materials, experimental systems and methods used in many studies. Here, indicate whether each material, system or method listed is relevant to your study. If you are not sure if a list item applies to your research, read the appropriate section before selecting a response.

Materials & experimental systems

n/a	Involved in the study	
☐	☒	Antibodies
☐	☒	Eukaryotic cell lines
☒	☐	Palaeontology and archaeology
☐	☒	Animals and other organisms
☒	☐	Clinical data
☒	☐	Dual use research of concern

Methods

n/a	Involved in the study	
☒	☐	ChIP-seq
☐	☒	Flow cytometry
☒	☐	MRI-based neuroimaging

Antibodies

Antibodies used

Antibodies for flow cytometry were obtained from Biolegend
For intracellular cytokine staining: Surface markers to identified CD4+ and CD8+ T cells including,
TCRβ APC/Cy7 (Clone: H57-597; Cat # 109219)
CD4 PE/Cy7 (Clone: RM4-4; Cat # 116015)
CD8 PreCP/Cy5.5 (Clone: 53–5.8; Cat # 140417)
CD44 BV605 (Clone: IM7; Cat # 563058)
Live/Dead marker (Cat # L34957)
Intracellular markers included: IFNγ Pacific Blue (Clone: XMG1.2; Cat # 505817), TNFα PE (Clone: MP6-XT22; Cat # 506305), Foxp3 Alex Fluor 488 (Clone: MF-14, Cat # 126405), IL2 PE/TexasRed.(Clone: JES6-5H4; Cat # 503839).

Validation

Validation is available on the vendor website (Biolegend).

Eukaryotic cell lines

Policy information about cell lines and Sex and Gender in Research

Cell line source(s)

RAW264.7 cells were from ATCC.

Authentication

No formal authentication was carried out. Cell morphology and adhesion was consistent with expectations.

Mycoplasma contamination

Cell lines were not tested for mycoplasma.

Commonly misidentified lines
(See ICLAC register)

No commonly misidentified cell lines were used in this study.

Animals and other research organisms

Policy information about studies involving animals; ARRIVE guidelines recommended for reporting animal research, and Sex and Gender in Research

Laboratory animals

6-week-old CD-1 mice, 6-week old C57BL/6 mice, New Zealand white rabbits.

Wild animals

This study did not involved wild animals.

Reporting on sex

For murine studies, female mice were used. For rabbit immunization studies, female and male rabbits were used as indicated.

Field-collected samples

This study did not involved Field-collected samples

Ethics oversight

All experiments involving mice in University at SUNY at Buffalo were carried out using protocols approved by SUNY at Buffalo Institutional Animal Care and Use Committee. All experiments involving mice in Ehime University were carried out using protocols approved by the Institutional Animal Care and Use Committee of Ehime University, and the experiments were conducted according to the Ethical Guidelines for Animal Experiments of Ehime University. Rabbit immunization was carried out by Kitayama labes (Ina, Japan) and by Pocono Rabbit Farm (Canadensis PA, USA) according to approved protocols.

Note that full information on the approval of the study protocol must also be provided in the manuscript.

Flow Cytometry

Plots

Confirm that:

☒ The axis labels state the marker and fluorochrome used (e.g. CD4-FITC).

☒ The axis scales are clearly visible. Include numbers along axes only for bottom left plot of group (a 'group' is an analysis of identical markers).

☒ All plots are contour plots with outliers or pseudocolor plots.

☒ A numerical value for number of cells or percentage (with statistics) is provided.

Methodology

Sample preparation	Splenocytes were harvested from the immunized mice on day 42. Spleens were collected and than passed through a 70 µm cell strainer in a 50 mL tube to collect single cells. Cells were centrifuged at 500 rcf, and red blood lysis buffer was added for 5 min on ice to lysed red blood cells. After incubation, 20 mL of PBS were added to dilute the lysis buffer, and samples were centrifuge at 500 rcf for 5 min. In 96-well culture plate, splenocytes were stimulated with 1 µl/ml of 230D1 or CSP for 18h, followed by incubation with brefeldin A for another 6 hr to block the cytokine secretion from the cells. Cells were stained for the surface markers using TCRβ APC/Cy7, CD4 PE/Cy7, CD8 PreCP/Cy5.5, CD44 BV605, Live/Dead marker diluted in FASC buffer (cold-PBS containing 0.5% BSA and 0.05% sodium azide) for 25 min on ice. The cells were washed with FASC buffer twice, then fixed with the fixation/permeabilization buffer for 10 min on ice. The cells were wash twice with FASC buffer, and permeabilization buffer were added into each well for 20 min on ice. Intracellular markers including IFNγ Pacific Blue, TNFα PE, Foxp3 Alex Fluor 488, IL2 PE/TexasRed.were diluted in permeabilization buffer, and cells were stained for 25 min on ice. Stained cells were washed twice with permeabilization buffer, then resuspended in FASC buffer.
Instrument	The instrument we use for sample collection is the BD LSRFortessa X-20.
Software	The software we use for flow analysis is FlowJo V10.
Cell population abundance	For triple cytokines T cells, there were 18.7% CD8+ cells and 23% CD4 + cells for both control and CoPoP group.<br>14% of CD44high cells in both CD4+ and CD8+ CoPoP group. And 0.3% of Triple cytokine cells in control. 7.8 % of triple cytokine cells in CoPoP group with Pfs230D1+ stimulation and 2.5% of triple cytokine cells in CoPoP group with CSP stimulation .
Gating strategy	For triple cytokines CD8 T cells: Cells were first gated with TCRbeta and CD8 (Fig S4). Then gated cells were identified based on surface marker in CD44high CD4 T cells, followed by gating IL2 positive cells, then gated with IFNgamma and TNFalph positive cells.<br>For triple cytokines CD4 T cells: Cells were first gated with TCRbeta and CD4 (Fig S5). Followed by Foxp3 negatived cells were collected. Then gated cells were identified based on surface marker in CD44high CD8 T cells, followed by gating IL2 positive cells, then gated with IFNgamma and TNFalph positive cells.

☒ Tick this box to confirm that a figure exemplifying the gating strategy is provided in the Supplementary Information.
